# Complete genome sequence of *Serratia marcescens* D1_6, isolated from peat soil

**DOI:** 10.1128/mra.00299-24

**Published:** 2024-09-23

**Authors:** Priscilla Isaac, Prasanna Mutusamy, Lee Su Yin, Yap Jing Wei, Faezah Mohd Salleh, Muhammad Abdul Latiff bin Abu Bakar, Sivachandran Parimannan, Heera Rajandas

**Affiliations:** 1 Centre of Excellence for Omics-Driven Computational Biodiscovery (COMBio), Aimst University, Bedong, Kedah, Malaysia; 2 Centre of Research for Sustainable Uses of Natural Resources (SUNR), Faculty of Applied Sciences and Technology (FAST), Universiti Tun Hussein Onn Malaysia, Pagoh Higher Education Hub, Muar, Johor, Malaysia; 3 Department of Biosciences, Faculty of Science, Universiti Teknologi Malaysia, Johor Bahru, Johor, Malaysia; 4 Environmental Management and Conservation Research Unit (eNCORe), Faculty of Applied Sciences and Technology (FAST), Universiti Tun Hussein Onn Malaysia, Pagoh Higher Education Hub, Muar, Johor, Malaysia; 5 Center for Evolutionary Hologenomics, Globe Institute, University of Copenhagen, Copenhagen, Denmark; The University of Arizona, Tucson, Arizona, USA

**Keywords:** *Serratia marcescens*, peat swamp forest, Illumina, Oxford Nanopore, hybrid assembly

## Abstract

We present a complete genome of *Serratia marcescens* D1_6 isolated from peat swamp forest. The complete genome for the isolate D1_6 was constructed using data from Oxford Nanopore Technologies and Illumina. The genome of D1_6 has a total length of 4,996,151 bp, comprising a chromosome and a plasmid.

## ANNOUNCEMENT

*Serratia marcescens*, a ubiquitous Gram-negative bacterium, is recognized as an opportunistic pathogen capable of causing nosocomial infections, including urinary tract and respiratory infections (1). Despite being primarily associated with opportunistic infections, this bacterium paradoxically possesses certain antimicrobial properties. It is known for its production of a bright red pigment known as prodigiosin, which demonstrates many promising potential therapeutic applications (2).

In this study, we report the complete genome of *Serratia marcescens* D1_6 strain, which was isolated in 2022 from peat soil collected from North Ayer Hitam Forest Reserve, Johor, Malaysia (N 02°04.5360’; E 102°49.0443’). One gram of soil was resuspended in 10 mL tryptic soy broth (TSB), followed by serial dilution and streak-plating on tryptic soy agar to obtain the strain. The isolate was aerobically cultivated in TSB medium at 30°C overnight prior to extraction. The same genomic DNA for both short- and long-read sequencing was extracted from a colony grown overnight using the NucleoSpin Tissue Kit (MACHEREY-NAGEL) following the manufacturer’s instructions.

Illumina libraries were prepared using the NEBNext Ultra II DNA Library Prep kit (NEB, USA) and sequenced on the NovaSeq 6000 platform using the 2 × 150 bp paired-end read strategy. A total of 4,493,088 paired-end reads (read length, 150 bp) were generated, which were then trimmed using Trimmomatic v0.39 (3) and subjected to quality control using fastQC (4) v0.12.1. Unsheared and non-size-selected DNA was used for the preparation of Nanopore libraries (SQK-NBD112.24 native barcoding kit, Oxford Nanopore Technologies) that were sequenced on the SpotON flow cell (R10.4.1) using a MinION device, and the basecaller used was Guppy v6.5.7 (high-accuracy model). The raw sequencing data of 94,232 reads with read length N₅₀ of 13,016 was trimmed using Porechop v0.2.4 (5), and the quality of reads was evaluated by Nanoplot v1.41.6 (6).

The hybrid *de novo* assembly with both short and long reads was performed using Unicycler v0.5.0 (7). All steps, including overlap identification, overlap trimming, and contig rotation, were done by Unicycler. The genome was rotated to begin with DNAA_SERMA (*dnaA*), while the plasmid was not rotated. The two contigs were confirmed to be a circular chromosome and a plasmid using Plassembler v1.5.0 (8). Genome annotation was carried out using the NCBI Prokaryotic Genome Annotation Pipeline (PGAP) v6.5 (9). Default parameters were used for all the software tools involved in the analysis otherwise specified.

The genome comprises 4,996,151 bp; it consists of one circular chromosome (4,991,506 bp) and one circular plasmid (4,645 bp). The assembled genome had a GC content of 59.72% (Table 1). A total of 4,755 genes made up of 4,614 intact coding sequences (CDS), 22 rRNAs, 88 tRNAs, 15 noncoding RNAs (ncRNAs), and 16 pseudogenes were detected in the genome. Antibiotic biosynthetic gene clusters (BGCs) of strain D1_6 were predicted using antiSMASH v7.0.0 (10), which uncovered secondary metabolite BCGs in 10 regions including one prodigiosin, betalactone, thiopeptide, RRE-containing, NRPS-like, opine-like-metallophore, NRP-metallophore, butyrolactone, and four NRPS. The presence of these diverse secondary metabolite BGCs suggests that strain D1_6 harbors the genetic potential to produce a wide array of bioactive compounds.

**TABLE 1:** Genome characteristics of *Serratia marcescens* D1_6

Characteristic	Value
Sequencing and assembly features	
No. of Illumina reads	4,493,088
No. of Nanopore reads	94,232
Total no. of Illumina bases	673,900,000
Total no. of Nanopore bases	706,563,571
Average genome coverage (×)	137
Genome features	
Genome size (bp)	4,991,506 bp
No. of contigs	2
GC content (%)	59.72
Total no. of genes	4,755
No. of coding sequences	4,614
No. of pseudogenes	16
No. of rRNAs	22
No. of tRNAs	88
No. of noncoding RNAs	15

## Data Availability

The complete genome sequences were deposited at NCBI GenBank under the BioProject accession number PRJNA949442, the Biosample accession number SAMN37190739, and the GenBank accession numbers CP133645 (genome) and CP133646 (plasmid). The raw reads were deposited in the Sequence Read Archive under the accession numbers SRR27205305 and SRR27194383.
